# *P*,*P*′-Diphenyl­ethyl­enediphosphinic acid dihydrate

**DOI:** 10.1107/S1600536812030954

**Published:** 2012-07-14

**Authors:** Charles D. Swor, Bryan P. Nell, Lev N. Zakharov, David R. Tyler

**Affiliations:** a Department of Chemistry, 1253 University of Oregon, Eugene, Oregon 97403-1253, USA

## Abstract

The title compound, C_14_H_16_O_4_P_2_·2H_2_O, possesses a crystallographic inversion center where two –P(=O)(OH)(C_6_H_5_) groups are joined together *via* two –CH_2_ groups. In the crystal, the acid molecules are linked by the water molecules *via* O—H⋯O hydrogen bonds, leading to the formation of a two-dimensional network lying parallel to (101).

## Related literature

For background on related phosphine macrocycles, see: Caminade & Majoral (1994 ▶); Swor & Tyler (2011 ▶). For related syntheses, see: Lambert & Desreux (2000 ▶). For literature related to the use of phosphine complexes as N_2_ scrubbers, see: Miller *et al.* (2002 ▶). For a related structure, see: Costantino *et al.* (2008 ▶). For literature related to the macrocycle effect, see: Melson (1979 ▶).
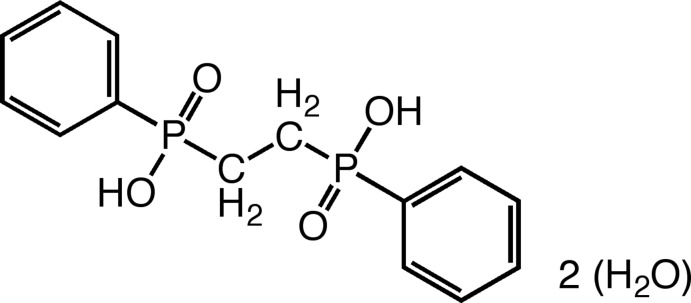


## Experimental

### 

#### Crystal data

C_14_H_16_O_4_P_2_·2H_2_O
*M* *_r_* = 346.24
Monoclinic, 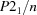

*a* = 10.8280 (16) Å
*b* = 6.2455 (10) Å
*c* = 12.861 (2) Å
β = 91.177 (2)°
*V* = 869.5 (2) Å^3^
*Z* = 2
Mo *K*α radiation
μ = 0.27 mm^−1^
*T* = 173 K
0.27 × 0.23 × 0.12 mm

#### Data collection

Bruker APEX CCD area-detector diffractometer
Absorption correction: multi-scan (*SADABS*; Bruker, 2000 ▶) *T*_min_ = 0.930, *T*_max_ = 0.968
9251 measured reflections
1888 independent reflections
1696 reflections with *I* > 2σ(*I*)
*R*_int_ = 0.021

#### Refinement

*R*[*F* ^2^ > 2σ(*F* ^2^)] = 0.039
*wR*(*F* ^2^) = 0.109
*S* = 1.09
1888 reflections
112 parameters
H atoms treated by a mixture of independent and constrained refinement
Δρ_max_ = 0.41 e Å^−3^
Δρ_min_ = −0.39 e Å^−3^

### 

Data collection: *SMART* (Bruker, 2000 ▶); cell refinement: *SAINT* (Bruker, 2000 ▶); data reduction: *SAINT*; program(s) used to solve structure: *SHELXTL* (Sheldrick, 2008 ▶); program(s) used to refine structure: *SHELXTL*; molecular graphics: *SHELXTL*; software used to prepare material for publication: *SHELXTL*.

## Supplementary Material

Crystal structure: contains datablock(s) I, global. DOI: 10.1107/S1600536812030954/bv2207sup1.cif

Structure factors: contains datablock(s) I. DOI: 10.1107/S1600536812030954/bv2207Isup2.hkl

Additional supplementary materials:  crystallographic information; 3D view; checkCIF report

## Figures and Tables

**Table 1:** Hydrogen-bond geometry (Å, °)

*D*—H⋯*A*	*D*—H	H⋯*A*	*D*⋯*A*	*D*—H⋯*A*
O2—H1*O*⋯O1*S*	1.06 (3)	1.40 (3)	2.459 (2)	173 (2)
O1*S*—H1*S*⋯O1^i^	0.87 (3)	1.82 (3)	2.687 (2)	178 (3)
O1*S*—H2*S*⋯O1^ii^	0.91 (4)	1.78 (4)	2.682 (2)	167 (3)

Symmetry codes: (i) 
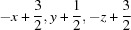
; (ii) 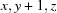.

**Fig. 1.**
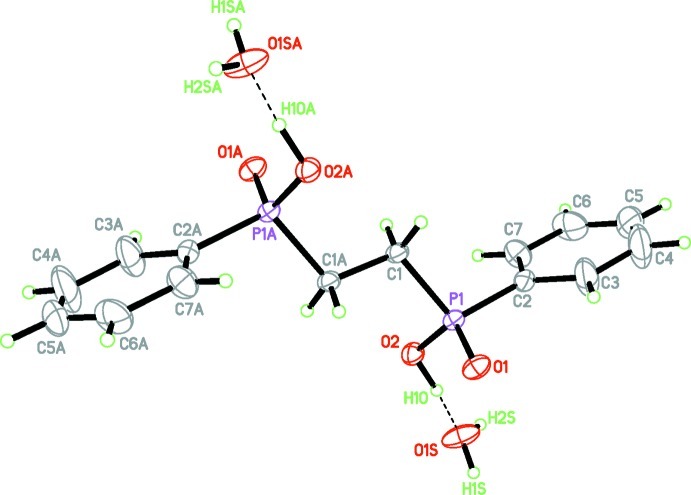
A fragment of the crystal structure of P,P'-diphenylethylenediphosphinic acid with 50% probability displacement ellipsoids and the atom-numbering scheme. [Symmetry code (A): -x,-y,-z].

## supplementary crystallographic information

### Comment

In a recent publication, we showed that complexes of the type *trans*-Fe(P_2_)_2_Cl_2_ (P_2_ = a bidentate phosphine) will react with dinitrogen at high pressure to form *trans*-[Fe(P_2_)_2_(N_2_)Cl]^+^ (Miller *et al.*, 2002). This reaction is potentially useful as a way to scrub dinitrogen from natural gas contaminated with dinitrogen. Unfortunately, the phosphine ligands in these dinitrogen-scrubbing complexes slowly dissociate in aqueous solution, leading to degradation of the complexes. This prevents a practical pressure-swing process from being developed. One potential method to obtain complexes that are more robust is to use a phosphine macrocycle in place of the two bidentate ligands. (The "macrocycle effect" predicts that the binding constant for a macrocyclic ligand is orders of magnitude higher than the binding constant for two bidentate ligands (Melson, 1979)).

In addition to their usefulness in the N_2_-scrubbing scheme described above, macrocyclic phosphine compounds are sought after in general as ligands for transition metal complexes because of their strong binding properties. However, the synthesis of phosphine macrocycles is a relatively underdeveloped area. One approach to macrocyclic phosphines is a template synthesis in which two secondary bidentate phosphines are coordinated to a common metal center and then covalently linked. The title molecule is both a precursor in the synthesis of the secondary bidentate phosphine 1,2-bis(phenylphosphino)ethane (MPPE, used in our laboratory for subsequent conversion into a macrocyclic phosphine ligand) and the oxidation product of MPPE. The X-ray structure of the title molecule recrystallized from ethanol has been reported (Costantino *et al.*, 2008). As might be expected, the structure has an extensive hydrogen bonding network involving oxygen atoms (in the P=O and –OH groups) and H atoms (in the O—H groups). In contrast to the method used in this previous report, the structure reported here was recrystallized from water, which resulted in a different structure due to solvent water molecules.

The title compound has a centrosymmetrical structure where two –P(=O)(OH)(C_6_H_5_) groups are joined together *via* two –CH_2_ groups. The terminal –OH group forms a very strong O(2)—H(1O)···O(1 s) H-bond with the solvent water molecule (Figure 1 and Table 1): the O(2)···(O1s), O(2)—H(10) and O(1 s)···H(10) distances are 2.459 (2), 1.06 (3) and 1.40 (3) Å, respectively and the O(2)—H(10)···O(1 s) angle is 173 (3)°.

### Experimental

The title molecule was prepared serendipitously while attempting to synthesize a phosphine macrocycle using a Cu(I) template. 1,2-Bis(phenylphosphino)ethane (MPPE) (2 equiv.) was reacted with Cu(MeCN)_4_PF_6_ (1 equiv.) in acetonitrile to yield the corresponding Cu(MPPE)_2_PF_6_ complex. A similar complex (with trifluoromethanesulfonate counter anion) was reported to be relatively air-stable for several months (Lambert & Desreux, 2000). However, after several weeks of exposure to air, the Cu(MPPE)_2_PF_6_ complex decomposed and the phosphine ligands were fully oxidized, yielding the title compound. The crude oxidized phosphine was recrystallized from water, yielding crystals of the title molecule. Note that the title compound can be reduced back to the starting secondary bis-phosphine.

### Refinement

The structure was solved using direct methods and refined with anisotropic thermal parameters for non-H atoms. H atoms in the main molecule were positioned geometrically and refined in a rigid group model, C—H = 1.2*U*_eq_(C) for –CH_2_ and –CH groups. H atoms in the terminal –OH group and in a solvent water molecule involved in intermolecular H-bonds were found from the residual density and refined with isotropic thermal parameters. There are some alongations of thermal parameters of the carbon atoms in the phenyl rings indicating that the phenyl rings in the structure are flexible.

### Crystal data

**Table:**

C_14_H_16_O_4_P_2_·2H_2_O	*F*(000) = 364
*M**_r_* = 346.24	*D*_x_ = 1.322 Mg m^−^^3^
Monoclinic, *P*2_1_/*n*	Mo *K*α radiation, λ = 0.71073 Å
Hall symbol: -P 2yn	Cell parameters from 4007 reflections
*a* = 10.8280 (16) Å	θ = 2.4–28.0°
*b* = 6.2455 (10) Å	µ = 0.27 mm^−^^1^
*c* = 12.861 (2) Å	*T* = 173 K
β = 91.177 (2)°	Block, colorless
*V* = 869.5 (2) Å^3^	0.27 × 0.23 × 0.12 mm
*Z* = 2	

### Data collection

**Table:**

Bruker APEX CCD area-detector diffractometer	1888 independent reflections
Radiation source: fine-focus sealed tube	1696 reflections with *I* > 2σ(*I*)
Graphite monochromator	*R*_int_ = 0.021
φ and ω scans	θ_max_ = 27.0°, θ_min_ = 2.4°
Absorption correction: multi-scan (*SADABS*; Bruker, 2000)	*h* = −13→13
*T*_min_ = 0.930, *T*_max_ = 0.968	*k* = −7→7
9251 measured reflections	*l* = −16→16

### Refinement

**Table:**

Refinement on *F*^2^	Primary atom site location: structure-invariant direct methods
Least-squares matrix: full	Secondary atom site location: difference Fourier map
*R*[*F*^2^ > 2σ(*F*^2^)] = 0.039	Hydrogen site location: inferred from neighbouring sites
*wR*(*F*^2^) = 0.109	H atoms treated by a mixture of independent and constrained refinement
*S* = 1.09	*w* = 1/[σ^2^(*F*_o_^2^) + (0.0533*P*)^2^ + 0.4488*P*] where *P* = (*F*_o_^2^ + 2*F*_c_^2^)/3
1888 reflections	(Δ/σ)_max_ < 0.001
112 parameters	Δρ_max_ = 0.41 e Å^−^^3^
0 restraints	Δρ_min_ = −0.39 e Å^−^^3^

### Special details

**Table:**

Geometry. All e.s.d.'s (except the e.s.d. in the dihedral angle between two l.s. planes)are estimated using the full covariance matrix. The cell e.s.d.'s are takeninto account individually in the estimation of e.s.d.'s in distances, anglesand torsion angles; correlations between e.s.d.'s in cell parameters are onlyused when they are defined by crystal symmetry. An approximate (isotropic)treatment of cell e.s.d.'s is used for estimating e.s.d.'s involving l.s. planes.
Refinement. Refinement of *F*^2^ against ALL reflections. The weighted *R*-factor *wR* and goodness of fit *S* are based on *F*^2^, conventional *R*-factors *R* are based on *F*, with *F* set to zero for negative *F*^2^. The threshold expression of *F*^2^ > σ(*F*^2^) is used only for calculating *R*-factors(gt) *etc*. and is not relevant to the choice of reflections for refinement. *R*-factors based on *F*^2^ are statistically about twice as large as those based on *F*, and *R*- factors based on ALL data will be even larger.

### Fractional atomic coordinates and isotropic or equivalent isotropic displacement parameters (Å^2^)

**Table:**

	*x*	*y*	*z*	*U*_iso_*/*U*_eq_	
P1	0.84023 (4)	0.19271 (7)	0.54585 (3)	0.02826 (16)	
O1	0.77895 (12)	0.0487 (2)	0.62175 (10)	0.0395 (3)	
O2	0.92075 (11)	0.3728 (2)	0.59502 (10)	0.0368 (3)	
C1	0.94816 (15)	0.0518 (3)	0.46715 (13)	0.0318 (4)	
H1B	0.9039	−0.0607	0.4270	0.038*	
H1C	0.9847	0.1527	0.4170	0.038*	
C2	0.72834 (16)	0.3127 (3)	0.45984 (14)	0.0356 (4)	
C3	0.6126 (2)	0.2218 (6)	0.4494 (2)	0.0835 (10)	
H3A	0.5926	0.0983	0.4887	0.100*	
C4	0.5256 (3)	0.3103 (9)	0.3818 (3)	0.1219 (18)	
H4A	0.4467	0.2452	0.3737	0.146*	
C5	0.5523 (3)	0.4894 (7)	0.3271 (2)	0.0949 (12)	
H5A	0.4910	0.5519	0.2827	0.114*	
C6	0.6673 (3)	0.5807 (5)	0.3356 (2)	0.0762 (8)	
H6A	0.6861	0.7047	0.2961	0.091*	
C7	0.7566 (2)	0.4916 (4)	0.40205 (17)	0.0526 (5)	
H7A	0.8366	0.5537	0.4076	0.063*	
O1S	0.8284 (2)	0.6587 (3)	0.69946 (14)	0.0622 (5)	
H1O	0.875 (2)	0.491 (4)	0.6401 (19)	0.062 (7)*	
H1S	0.792 (3)	0.621 (5)	0.756 (3)	0.086 (10)*	
H2S	0.801 (3)	0.783 (6)	0.670 (3)	0.092 (10)*	

### Atomic displacement parameters (Å^2^)

**Table:**

	*U*^11^	*U*^22^	*U*^33^	*U*^12^	*U*^13^	*U*^23^
P1	0.0299 (2)	0.0269 (3)	0.0281 (2)	0.00257 (16)	0.00540 (16)	0.00037 (15)
O1	0.0459 (7)	0.0341 (7)	0.0390 (7)	0.0028 (6)	0.0142 (6)	0.0059 (5)
O2	0.0347 (6)	0.0363 (7)	0.0395 (7)	0.0003 (5)	0.0030 (5)	−0.0077 (6)
C1	0.0347 (9)	0.0328 (9)	0.0280 (8)	0.0056 (7)	0.0050 (7)	−0.0023 (7)
C2	0.0308 (9)	0.0425 (10)	0.0337 (9)	0.0061 (7)	0.0022 (7)	0.0010 (7)
C3	0.0406 (12)	0.131 (3)	0.0778 (18)	−0.0223 (16)	−0.0133 (12)	0.0428 (19)
C4	0.0413 (14)	0.226 (5)	0.097 (2)	−0.011 (2)	−0.0203 (15)	0.066 (3)
C5	0.0592 (17)	0.166 (4)	0.0587 (16)	0.044 (2)	−0.0078 (13)	0.028 (2)
C6	0.103 (2)	0.0722 (18)	0.0535 (14)	0.0296 (17)	−0.0077 (14)	0.0196 (13)
C7	0.0597 (13)	0.0459 (12)	0.0518 (12)	0.0027 (10)	−0.0082 (10)	0.0102 (10)
O1S	0.1045 (15)	0.0336 (8)	0.0501 (9)	0.0127 (8)	0.0378 (10)	0.0049 (7)

### Geometric parameters (Å, º)

**Table:**

P1—O1	1.4928 (13)	C3—H3A	0.9500
P1—O2	1.5500 (13)	C4—C5	1.355 (5)
P1—C2	1.7883 (18)	C4—H4A	0.9500
P1—C1	1.7927 (16)	C5—C6	1.373 (5)
O2—H1O	1.06 (3)	C5—H5A	0.9500
C1—C1^i^	1.534 (3)	C6—C7	1.393 (3)
C1—H1B	0.9900	C6—H6A	0.9500
C1—H1C	0.9900	C7—H7A	0.9500
C2—C7	1.380 (3)	O1S—H1O	1.40 (3)
C2—C3	1.380 (3)	O1S—H1S	0.87 (3)
C3—C4	1.383 (4)	O1S—H2S	0.91 (4)
			
O1—P1—O2	115.11 (8)	C2—C3—H3A	119.9
O1—P1—C2	110.63 (8)	C4—C3—H3A	119.9
O2—P1—C2	108.45 (8)	C5—C4—C3	120.4 (3)
O1—P1—C1	112.15 (8)	C5—C4—H4A	119.8
O2—P1—C1	102.64 (8)	C3—C4—H4A	119.8
C2—P1—C1	107.32 (8)	C4—C5—C6	120.3 (2)
P1—O2—H1O	117.6 (14)	C4—C5—H5A	119.9
C1^i^—C1—P1	111.97 (15)	C6—C5—H5A	119.9
C1^i^—C1—H1B	109.2	C5—C6—C7	120.0 (3)
P1—C1—H1B	109.2	C5—C6—H6A	120.0
C1^i^—C1—H1C	109.2	C7—C6—H6A	120.0
P1—C1—H1C	109.2	C2—C7—C6	119.7 (2)
H1B—C1—H1C	107.9	C2—C7—H7A	120.2
C7—C2—C3	119.5 (2)	C6—C7—H7A	120.2
C7—C2—P1	121.16 (15)	H1O—O1S—H1S	116 (2)
C3—C2—P1	119.37 (18)	H1O—O1S—H2S	122 (2)
C2—C3—C4	120.1 (3)	H1S—O1S—H2S	116 (3)
			
O1—P1—C1—C1^i^	−60.33 (19)	C7—C2—C3—C4	−0.3 (5)
O2—P1—C1—C1^i^	63.78 (18)	P1—C2—C3—C4	−178.9 (3)
C2—P1—C1—C1^i^	177.97 (16)	C2—C3—C4—C5	−1.5 (6)
O1—P1—C2—C7	162.61 (16)	C3—C4—C5—C6	2.2 (6)
O2—P1—C2—C7	35.49 (19)	C4—C5—C6—C7	−1.2 (5)
C1—P1—C2—C7	−74.74 (18)	C3—C2—C7—C6	1.3 (4)
O1—P1—C2—C3	−18.8 (3)	P1—C2—C7—C6	179.83 (19)
O2—P1—C2—C3	−146.0 (2)	C5—C6—C7—C2	−0.6 (4)
C1—P1—C2—C3	103.8 (2)		

Symmetry code: (i) −*x*+2, −*y*, −*z*+1.

### Hydrogen-bond geometry (Å, º)

**Table:**

*D*—H···*A*	*D*—H	H···*A*	*D*···*A*	*D*—H···*A*
O2—H1*O*···O1*S*	1.06 (3)	1.40 (3)	2.459 (2)	173 (2)
O1*S*—H1*S*···O1^ii^	0.87 (3)	1.82 (3)	2.687 (2)	178 (3)
O1*S*—H2*S*···O1^iii^	0.91 (4)	1.78 (4)	2.682 (2)	167 (3)

Symmetry codes: (ii) −*x*+3/2, *y*+1/2, −*z*+3/2; (iii) *x*, *y*+1, *z*.

## References

[bb1] Bruker (2000). *SMART*, *SAINT* and *SADABS* Bruker AXS Inc., Madison, Wisconsin, USA.

[bb2] Caminade, A.-M. & Majoral, J. P. (1994). *Chem. Rev.* **94**, 1183–1213.

[bb3] Costantino, F., Ienco, A., Midollini, S., Orlandini, A., Sorace, L. & Vacca, A. (2008). *Eur. J. Inorg. Chem.* pp. 3046–3055.

[bb4] Lambert, B. & Desreux, J. F. (2000). *Synthesis*, **12**, 1668–1670.

[bb5] Melson, G. (1979). In *Coordination Chemistry of Macrocyclic Compounds.* New York: Plenum Press.

[bb6] Miller, W. K., Gilbertson, J. D., Leiva-Paredes, C., Bernatis, P. R., Weakley, T. J. R., Lyon, D. K. & Tyler, D. R. (2002). *Inorg. Chem.* **41**, 5453–5465. 10.1021/ic025774q 12377040

[bb7] Sheldrick, G. M. (2008). *Acta Cryst.* A**64**, 112–122. 10.1107/S0108767307043930 18156677

[bb8] Swor, C. D. & Tyler, D. R. (2011). *Coord. Chem. Rev.* **255**, 2860–2881.

